# The independent association between diet quality and body composition

**DOI:** 10.1038/srep04928

**Published:** 2014-05-12

**Authors:** Clemens Drenowatz, Robin P. Shook, Gregory A. Hand, James R. Hébert, Steven N. Blair

**Affiliations:** 1 Department of Exercise Science, Arnold School of Public Health, University of South Carolina, Columbia, South Carolina 29208, USA; 2 South Carolina Statewide Cancer Prevention and Control Program, Arnold School of Public Health, University of South Carolina, Columbia, South Carolina 29208, USA; 3 Department of Epidemiology and Biostatistics, Arnold School of Public Health, University of South Carolina, Columbia, South Carolina 29208, USA

## Abstract

Excess body weight is associated with an imbalance between energy expenditure and dietary intake but evidence on the association between diet quality and body composition remains equivocal. Rather than relying on differences in diet quality between overweight/obese and normal weight adults, this study examined the association between the Healthy Eating Index 2010 (HEI-2010) and body fatness on a continuous scale, independent of physical activity (PA). Further the association between components of the HEI-2010 and risk for overweight/obesity was explored. 407 adults (27.6 ± 3.7 years) provided at least two 24-hour diet recalls over a period of 14 days, which were used to calculate the HEI-2010. Percent body fat (BF) was assessed via dual X-ray absorptiometry and PA was determined via a multi-sensor device, worn over a period of 10 days. PA was a stronger contributor to the variability in BF than the HEI-2010 and the association between HEI-2010 and BF was significant only in men. Particularly a high consumption of protein, sodium and empty calories increased the risk for overweight/obesity. Adherence to dietary guidelines positively affects body fatness in men, independent of PA. In contrast to current dietary recommendations, the risk for overweight/obesity was increased with a higher protein intake.

Excessive body weight has been associated with various chronic diseases, including cardiovascular disease, diabetes, many forms of cancer, and numerous musculoskeletal problems^1^,^2^. Thus, the World Health Organization considers obesity as one of the leading future threats to public health^3^. In the United States 69% of adults are considered to be overweight or obese^4^, causing an estimated US$ 147 billion in medical costs^5^. In order to address excess body weight and high body fatness, sufficient physical activity (PA) with a balanced diet and consumption of nutrient-dense foods has been emphasized^6^. Several studies have shown that a higher intake of fruits, vegetables, and whole grains is associated with lower BMI^7^,^8^. In addition to the examination of associations of specific food groups on body composition, various composite scores have been developed to determine overall diet quality. The Healthy Eating Index (HEI), for example, is a measure of conformance with the “Dietary Guidelines for Americans” (DGA)^9^. It provides a single continuous score ranging from 0 to 100, with higher scores reflecting a closer adherence to dietary recommendations, which would indicate a better diet quality. Higher scores for HEI as well as other dietary indices have been associated with a risk reduction for cardiovascular disease and diabetes^10^,^11^. There also is some evidence on the beneficial effects of adherence to dietary guidelines and the risk for overweight and obesity^11^,^12^,^13^. Other studies, however, do not provide clear evidence of protective effects of DGA-like patterns and weight gain^8^,^14^,^15^.

Differences in methodology and the accuracy of the assessment of dietary intake may partially explain the equivocal results. Most studies relied on food frequency questionnaires^12^,^13^,^14^,^15^,^16^,^17^, which tend to have a low correlation with true diet and, therefore, are more prone to measurement error^18^,^19^. In addition, the assessment and inclusion of potentially confounding variables needs to be considered. The association between diet and PA or sedentary behavior necessitates the inclusion of PA as a covariate when examining the association between diet quality and body composition^20^,^21^. Previous studies, however, relied on self-reported PA rather than on objective measurements^12^,^13^,^14^,^15^,^16^,^17^. The utilization of a continuous measure of body composition, rather than focusing solely on differences between overweight or obese versus normal weight participants would enhance the understanding of the association between diet quality and body composition. Further, there is a lack of research on differences in the association between diet, PA and body composition in men and women. Health behaviors, however, differ between sexes; men are generally more active than women^22^, while women, in general, consume a healthier diet^13^,^23^. Additionally, there are important differences in self-report biases by sex^24^. These differences could affect the association between diet quality and body composition. Lassalle et al. showed a stronger association between adherence to dietary guidelines and body composition in men compared to women in French adults^23^ but these authors did not consider potential differences in self-reported bias and relied on self-reported PA.

The present study utilizes objective measurements of PA along with measures of social desirability and social approval to address potential bias in self-reported dietary intake and utilizes percent body fat in addition to body mass index (BMI) as indicator for body composition, which provides additional information on the relationship between diet quality, PA and body composition. Specifically, the purpose of this study was to examine the association between diet quality and body composition on a continuous scale separately for men and women while considering objectively determined PA. As the publication of the DGA-2010^6^ necessitated an adaptation of the HEI (HEI-2010)^25^, the association between the HEI-2010 and its components with the risk for overweight and obesity was examined as well.

## Results

Participants included in the analysis did not differ in ethnicity or educational background compared to those excluded due to lack of compliance. There were also no differences in anthropometric characteristics. Table 1 provides an overview of the sample characteristics of participants included in the subsequent analyses. 2/3 of the sample (66.1%) were European American with the majority of participants (83.8%) having 4 or more years of college education. Significantly more women (90.0%) than men (77.3%) had a college degree while ethnic distribution did not differ between men and women. There were no underweight participants and 31.9% and 15.2% were classified as overweight and obese, respectively. The prevalence of overweight was higher in men compared to women (39.4% vs. 24.9%) while the reverse was observed for obesity (11.1% vs. 19.1%). The combined prevalence of overweight and obesity did not differ between men and women and there was no difference between men and women for body mass index (BMI, 25.7 ± 3.4 vs. 25.5 ± 4.3).

The average HEI-2010 score was 60.3 with only 12 participants reporting a good diet quality (HEI > 80). A poor diet quality (HEI < 51) was reported by 85 participants. HEI-2010 scores were lower in men compared women (F (1, 405) = 8.67, p < .01). Men, however, reported a higher absolute and relative EI compared to women (F_abs_ (1, 405) = 94.89, p < .01; F_rel_ (1, 405) = 13.03, p < .01). Men also spent more time in moderate-to-vigorous PA (MVPA) compared to women (F (1, 405) = 39.44, p < .01), while no sex differences were observed for sedentary time (F (1, 405) = .52, p = .47).

After controlling for sex, age, education and ethnicity, a low, but significant correlation of HEI-2010 as well as energy intake (EI, kcal/day) and percent body fat (BF) was observed (Table 2). Correlations between MVPA or sedentary time and BF were more pronounced with highest correlations between MVPA and BF. In the sex-specific analyses, the associations between dietary variables and BF remained significant only in men and the relationship between MVPA or sedentary time and BF was stronger in women compared to men. MVPA further correlated with HEI-2010 in women and EI in men. Due to the high correlation between MVPA and sedentary time, only one of these parameters was entered into the linear regression analysis at a time. As was shown in the correlation analyses, MVPA was the strongest coefficient regarding BF (Table 3). HEI-2010 contributed significantly to the regression model in men, while in women only MVPA or sedentary time remained significant. Nevertheless, a greater portion of the variance in BF was explained in women compared to men.

Logistic regression, adjusted for age, education and ethnicity, revealed a significant inverse association between the risk for overweight/obesity and HEI-2010 in both men (OR = 0.97; CI: 0.94; 0.99) and women (OR = 0.96; CI: 0.94; 0.99). After additionally controlling for MVPA, a significant risk reduction for overweight and obesity with higher HEI-2010 scores remained only in men (Table 4). Specifically a lower sodium intake and a lower consumption of empty calories were associated with a reduced risk for overweight/obesity. A higher protein intake, on the other hand, was associated with an increased risk for overweight/obesity even though a higher protein intake would increase the HEI-2010 score. In women, only sodium intake was associated with a significant reduction for overweight/obesity. Additionally adjusting for social desirability and social approval did not affect any of the previously reported results.

## Discussion

The present findings show a significant but low correlation between body composition and diet quality, which is consistent with studies using previous versions of the HEI^11^,^12^,^13^. MVPA, however, seems to have a stronger effect on body composition compared to sedentary behavior and diet quality. Of particular interest are the differences in the effect of diet quality on body composition and risk for overweight/obesity between men and women. After adjusting for MVPA, the association between BF or overweight/obesity and HEI-2010 remained only in men. These results are in accordance with a more pronounced association between adherence to nutritional guidelines and markers of adiposity in men compared to women in a French population^23^. The lack of significance for HEI-2010 in women may be due to higher HEI-2010 scores in women compared to men. Higher HEI-2010 scores could reflect their greater emphasis on a healthy diet^26^, which may have resulted in a greater reporting bias in women. Results, however, remained after adjusting for social desirability and social approval. Higher MVPA levels in men compared to women could further have contributed to differences in the association between HEI-2010 and BF, as a more pronounced effect of diet quality on body composition has been shown with higher PA^27^.

The benefits of adherence to dietary guidelines could be explained by the importance of macronutrient balance for the regulation of various biological processes that are associated with cardiovascular disease risk and body composition^28^. Protein intake is particularly emphasized by the HEI-2010 as it has been argued that humans prioritize the absolute intake of protein over total energy needs^28^. A low protein content in the diet, therefore, could cause an increase in energy intake in order to meet protein requirements. Results of the present study, however, show an increased risk for overweight/obesity with high protein intake. A similar association has also been reported in a large U.S. sample^29^. These findings may be explained by the fact that people consume foods rather than nutrients and that there is a positive correlation between fat and total protein intake (results not shown). The replacement of some meat and poultry with other protein containing foods has been recommended^25^ and the HEI-2010 has included a separate category for seafood and plant-based protein, which suggests the awareness of potential detrimental effects of a high animal-based protein intake. Rather than relying on absolute protein intake, the utilization of a ratio of total protein intake to seafood and plant based protein may be a better indicator for a healthy diet. Another option could be to consider the ratio between total protein and fat intake. In addition to fat content, meat products have been shown to be one of the major sources of sodium^30^. The association between sodium, meat and fat consumption (results not shown) could also explain the higher risk for overweight/obesity with a high sodium intake. In addition, sodium intake is associated with a high consumption of processed foods^31^,^32^, which are generally of higher energy density. Consistent with findings of the present study a higher consumption of energy dense foods, which would be indicated by the amount of empty calories, increases the risk for passive overconsumption^32^,^33^,^34^. Besides the link between sodium intake and EI, a high sodium intake has been shown to cause adipocyte hypertrophy due to an alteration in adipocyte insulin sensitivity^35^,^36^.

Overall, the association between body composition and MVPA, however, seems to be stronger than that between body composition and diet quality. This may partially be explained by the generally high levels of MVPA and relatively low HEI-2010 scores in the present sample. It may be necessary to achieve a better adherence to dietary recommendations than that observed in the present study to experience positive effects of diet quality on body composition. The stronger association between MVPA and body composition may also be due to a more accurate assessment of PA compared to dietary intake. Limitations of self-reported intake have been well documented.^37^,^38^ The utilization of a comprehensive diet quality score and multiple 24 HR, however, has been shown to be less prone to measurement error compared to total EI^18^. As the HEI is calculated relative to total caloric intake the information may still accurately reflect dietary patterns even if total dietary intake is under- or over-reported^15^. A more stringent inclusion criteria for dietary reports of ±1 standard deviation in the ratio of energy intake to energy expenditure to determine plausible dietary reports^39^, did not significantly alter the results of the present study either (results not shown). Further, potential biases due to social desirability and social approval were considered in the analysis adding credibility to the results of this study. Nevertheless, selective misreporting of dietary intake needs to be considered as participants may purposely omit high fat snacks, alcohol or other foods that are considered unhealthy. There was also no differentiation between weekdays and weekend days on dietary reports even though there may be a difference in diet quality in different days of the week.

The cross-sectional design of the study also needs to be considered when interpreting the findings. Health behaviors were assessed over a period of 10 to 14 days and provide only a snapshot of these behaviors even though participants were screened for changes in body weight or health behavior leading up to the study. As the study population consisted predominantly of European-American adults with a college degree, the generalizability of the results may be limited. The proportion of participants classified as needing improvement in their diet, however, was similar to that reported in a large, representative US-sample^13^ while the prevalence of overweight/obesity was lower than in the general U.S. population^4^. The lower prevalence of overweight/obesity may actually strengthen the reported association between diet quality and body composition as it indicates a relationship between diet quality and body composition in healthy weight as well as overweight/obese subjects^13^. The utilization of continuous scores for HEI, PA and body composition also supports this argument.

In summary, this study provides evidence of an association between diet quality and body composition independent of PA, particularly in men. Specifically a lower intake of sodium and empty calories has been shown to positively affect body composition while a high protein intake has been shown to increase the risk for overweight/obesity. The inverse relationship between protein intake and body composition should be emphasized as a high protein consumption is a popular recommendation for weight loss^40^. Based on the present findings and results of a recent study including a large US representative sample^29^ the emphasis on high protein content needs to be revisited. Rather than total protein intake, more emphasis on lean protein sources may be necessary. Results of the present study also indicate a stronger effect of PA on body composition compared to diet quality. Due to their lower PA levels, women may obtain greater benefits by increasing their MVPA, while the lower diet quality of men may make them more susceptible to the benefits of an increase in diet quality. Most likely a combination of a balanced diet and sufficient PA will provide the greatest benefits but more research is needed to determine the differential effects of diet and PA on body composition and health in different populations and at different age range.

## Methods

### Study Sample

Baseline data from an observational study, including 430 (49.3% male) adults between 21 and 35 years of age was used as this age group is at particular risk for increasing weight and percent body fat^41^. Specifics of the study have been described previously^42^. As only participants with at least two 24-hour diet recalls (24HR) and at least 7 days (including 2 weekend days) of PA data were included in the present analysis the final sample size consists of 407 (48.6% male) adults. All participants signed an informed consent prior to data collection. The study was approved by the University of South Carolina Institutional Review Board and is in accordance with the Declaration of Helsinki.

### Dietary assessment

Multiple 24HR interviews were administered over the phone by a team of experienced registered dietitians over a period of 14 days. Despite the limitations of the reliance on self-report this method is considered the most accurate currently available tool for the assessment of dietary intake in a natural environment^43^. In order to increase accuracy of dietary reports, participants were given a validated 2-dimensional food portion visual^44^ and received 10–15 minutes of training on how to use it to estimate portion sizes of commonly eaten foods. Dietary data were analyzed using the Nutrient Data System for Research software (NDSR Version2012; Nutrition Coordinating Center, University of Minnesota, Minneapolis, MN, USA). Average values from the multiple 24HR were used to calculate the HEI-2010^25^. The rationale and significance of the utilization of the HEI as measure of diet quality has been documented previously^45^. The HEI-2010 consists of 10 food components and 2 nutrients (total fruit, whole fruit, total vegetable, greens and beans, whole grains, dairy products, total protein, seafood and plant-based protein, fatty acids; moderation: refined grains, sodium, empty calories). Empty calories, defined as calories from solid fats, added sugars and alcohol above a threshold of 13 grams/1000 kcal, refined grains and sodium are referred to as categories of moderation as a higher score is associated with a lower consumption. In all other categories, a higher score reflects a higher consumption of the respective components. In order to adjust for total EI components are scored relative to caloric density (per 1000 kcal). The individual components are summed to provide a single score between 0 and 100 with higher scores indicating a higher conformance with current dietary guidelines^25^. For the original HEI, scores above 80 indicate a good diet, while scores below 51 reflect a poor diet. A HEI score between 51 and 80 is considered as needing dietary improvement^46^.

### Assessment of energy expenditure and physical activity

Energy expenditure was measured with the SenseWear Armband (SWA, BodyMedia® Inc., Pittsburgh, PA. USA). The SWA incorporates tri-axial accelerometry, galvanic skin response, heat flux, skin temperature, and near body temperature; and has been shown to provide accurate estimates of energy expenditure in free-living adults^47^,^48^. Subjects were asked to wear the armband for 24 hours over a period of 10 days. This was during the same time frame the 24HR were administered. In order to be included in the analysis 7 days (including two weekend days) with at least 18 hours of wear time/day needed to be available. During periods of non-wear time participants recorded their activities, which were subsequently used to implement energy expenditure based on the 2011 Compendium of Physical Activity^49^. MET values per minute were used to determine time spent sedentary (sedentary < = 1.5 METs) in light (1.5 < light < 3.0 METs) and in MVPA (MVPA > = 3.0 METS).

### Anthropometric measurements and confounding variables

Body weight (kg) and height (cm) were measured with participants dressed in surgical scrubs and in bare feet according to standard procedures. Body weight was measured to the nearest 0.1 kg using an electronic scale (Healthometer® model 500 KL, McCook, IL, USA) and height was measured to the nearest 0.1 cm using a wall-mounted stadiometer (Model S100, Ayrton Corp., Prior Lake, MN, USA). The average of 3 measurements was used to calculate body mass index (kg/m^2^), which was subsequently used to differentiate between normal weight (18.5 < BMI < 25) and overweight/obese subjects (BMI ≥ 25)^50^. Fat mass and fat free mass were measured via a Lunar fan-beam dual X-ray absorptiometry (DXA) scanner (GE Healthcare model 8743, Waukesha, WI, USA) and used to calculate BF. Demographic information including age, education, and ethnicity, were obtained via questionnaire. Participants also completed the Marlow-Crowne Social Desirability Scale^51^ and the Martin-Larsen Approval Motivation scale^52^ as social desirability and social approval have been shown to affect self-reported dietary information^19^.

### Statistical analysis

Differences between men and women were examined by ANOVA for continuous variables or Chi-square tests for nominal variables. Subsequent analyses were performed for the total sample adjusting for sex and separately for men and women. Partial correlation, additionally adjusting for age, education and ethnicity, was used to examine the association between BF, HEI-2010, time spent in MVPA, and time spent sedentary. This analysis further assessed the potential risk of multicollinearity (r > 0.7/r < −0.7) in the subsequent regression analyses. Linear regression analysis was used to determine the combined association of HEI-2010, MVPA, and sedentary time with BF including the previously mentioned covariates. In a second analysis social desirability and social approval were added as additional covariates to address the potential of dietary misrepresentation. Logistic regression was carried out in a similar manner to determine the association between diet quality and the risk for overweight/obesity. All analyses were carried out with SPSS® 21.0 (SPSS Inc., Chicago, IL, USA) with a significance level of α = 0.05.

## Figures and Tables

**Table 1:** Descriptive characteristics. Values are mean ± SD (except for ethnicity and education where prevalence is reported)

	Male		Female	
	Normal Weight N = 98	Overweight/Obese N = 100	Normal Weight N = 117	Overweight/Obese N = 92
**% Eur. American**	66.3%	68.0%	74.5%	53.3%
**% College degree**	73.5%	81.0%	92.3%	87.0%
**Age (years)**	26.3 ± 3.5	28.5 ± 3.8	27.6 ± 3.4	28.1 ± 3.9
**Height (cm)**	178.7 ± 6.9	177.7 ± 7.0	165.1 ± 6.4	164.7 ± 6.2
**Weight (kg)**	73.2 ± 7.2	89.2 ± 11.4	60.5 ± 6.4	80.3 ± 10.0
**BMI (kg/m^2^)**	22.9 ± 1.4	28.2 ± 2.6	22.2 ± 1.6	29.7 ± 2.8
**% body fat**	17.0 ± 6.9	24.8 ± 7.9	30.0 ± 6.4	41.2 ± 5.3
**HEI-2010 score**	60.3 ± 13.1	57.0 ± 11.2	63.8 ± 10.3	59.7 ± 10.9
**Energy Intake (kcal/kg/day)**	33.3 ± 9.8	26.4 ± 8.4	30.2 ± 7.8	22.2 ± 5.8
**Time MVPA (min/day)**	193.6 ± 80.6	123.6 ± 64.5	141.3 ± 64.1	75.8 ± 47.1
**Time Sedentary (min/day)**	1 063.4 ± 95.9	1 106.1 ± 84.9	1 073.4 ± 81.4	1 113.9 ± 81.0

**Table 2:** Correlation coefficients, adjusted for sex (in total sample), age, race/ethnicity and education in men and women. Values are correlation coefficients (p-value)

TOTAL SAMPLE	HEI-2010	EI (kcal/day)	MVPA (min/day)	Sedentary (min/day)
**% body fat**	−.177 (<.001)	−.201 (<.001)	−.573 (<.001)	.392 (<.001)
**HEI score**		.055 (.267)	.0116 (.020)	−.043 (.390)
**EI (kcal/day)**			.146 (.003)	−.189 (<.001)
**MVPA (min/day)**				−.774 (<.001)
**MEN ONLY**	HEI-2010	EI (kcal/day)	MVPA (min/day)	Sedentary (min/day)
**% body fat**	−.204 (.004)	−.303 (<.001)	−.445 (<.001)	.290 (<.001)
**HEI score**		.143 (.046)	.094 (.191)	−.003 (.967)
**EI (kcal/day)**			.189 (.008)	−.232 (.001)
**MVPA (min/day)**				−.822 (<.001)
**WOMEN ONLY**	HEI-2010	EI (kcal/day)	MVPA (min/day)	Sedentary (min/day)
**% body fat**	−.154 (.027)	.069 (.324)	−.708 (<.001)	.485 (<.001)
**HEI score**		−.066 (.347)	.166 (.017)	−.097 (.165)
**EI (kcal/day)**			.079 (.259)	−.134 (.055)
**MVPA (min/day)**				−.714 (<.001)

**Table 3:** Standardized coefficients based on linear regression for % body fat, adjusted for sex (in total sample) age, race/ethnicity and education

	HEI-2010	MVPA time (min/day)	Sedentary time (min/day)	R^2^
**TOTAL SAMPLE**	−.086 **	−.454 **		.632
	−.124 **		.296 **	.541
**MEN ONLY**	−.164 *	−.451 **		.273
	−.204 **		.295 **	.180
**WOMEN ONLY**	−.038	−.699 **		.512
	−.108		.476 **	.260

*sig. coefficient (p < 0.05).

**sig. coefficient (p < 0.01).

**Table 4:** Odds ratios and 95% Confidence Intervals for overweight/obesity for HEI-2010 and the component scores. Values are adjusted for sex (total sample), age, education, race/ethnicity and time spent in MVPA

	Total Sample	Men	Women
**HEI-2010**	0.97 [0.95; 0.99]	0.97 [0.94; 0.99]	0.98 [0.94; 1.01]
**Component Scores**^1^			
Fruit	0.91 [0.75; 1.10]	0.85 [0.63; 1.16]	0.90 [0.68; 1.18]
Whole Fruit	1.00 [0.83; 1.20]	1.23 [0.91; 1.68]	0.96 [0.75; 1.24]
Vegetable	0.84 [0.63; 1.12	0.72 [0.47; 1.09]	1.00 [0.63; 1.58]
Greens and Beans	1.01 [0.89; 1.16]	0.96 [0.79; 1.17]	1.03 [0.85; 1.25]
Whole Grains	0.98 [0.91; 1.07]	1.03 [0.91; 1.17]	0.97 [0.86; 1.09]
Dairy	1.00 [0.90; 1.10]	0.90 [0.77; 1.07]	1.07 [0.93; 1.24]
Protein	1.89 [1.20; 2.97]	4.66 [2.10; 10.33]	1.06 [0.65; 2.01]
Seafood & Plant Protein	0.81 [0.61; 1.06]	0.83 [0.53; 1.31]	0.79 [0.53; 1.17]
Fatty Acid	1.02 [0.92; 1.12]	1.02 [0.88; 1.18]	1.07 [0.91; 1.25]
Refined Grains *	1.02 [0.92; 1.12	0.94 [0.81; 1.01]	1.03 [0.90; 1.19]
Sodium *	0.84 [0.76; 0.93]	0.78 [0.66; 0.93]	0.86 [0.74; 0.99]
Empty Calories *	0.93 [0.86; 1.02]	0.84 [0.73; 0.97]	0.97 [0.85; 1.10]

^1^all HEI-2010 component scores entered simultaneously.

*moderation categories: low intake results in higher HEI score.
